# Satellite artifacts modulate FireCCILT11 global burned area

**DOI:** 10.1038/s41467-024-46168-0

**Published:** 2024-03-08

**Authors:** Louis Giglio, David P. Roy

**Affiliations:** 1 Department of Geographical Sciences, University of Maryland, College Park, MD USA; 2 Center for Global Change and Earth Observations, and Department of Geography, Environment, & Spatial Sciences, Michigan State University, East Lansing, MI USA

**Keywords:** Climate sciences, Natural hazards

**arising from** A. Cardil et al. *Nature Communications* https://doi.org/10.1038/s41467-023-36052-8 (2023)

Climate teleconnections (CTs) remotely influence weather conditions and so may influence fire activity. A recent study by Cardil et al. ^1^ (hereafter C2023) reported relationships between burned area (BA) documented using the 1982–2018 FireCCILT11 BA product^2^, which is derived from NOAA Advanced Very High Resolution Radiometer (AVHRR) satellite imagery, and major CTs. Critically, C2023 did not evaluate the impact of known FireCCILT11 flaws on their findings, including in regions where they found significant relationships but where the FireCCILT11 BA time series is spurious. The resulting regional CT-fire relationships reported worldwide by C2023 are consequently open to question.

**Fig. 1:**
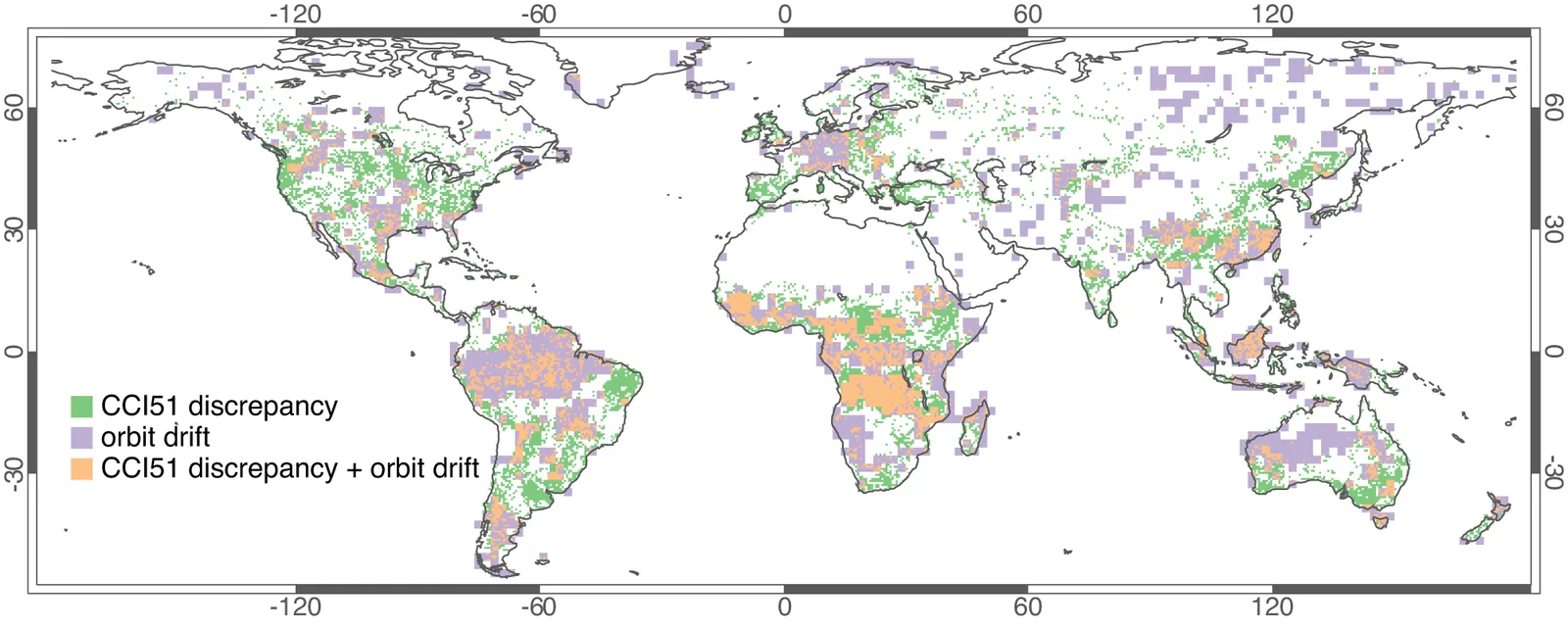
Global map of regions in which the FireCCILT11 product exhibits major satellite orbit-drift artifacts (“orbit drift”) and/or very poor agreement with the FireCCI51 reference burned area (BA) data set used for training and calibration (“CCI51 discrepancy”). The specific criteria used to identify these locations was as follows. Orbit drift: Spearman rank-order correlation between 1982–2000 FireCCILT11 total annual BA and mid-year AVHRR solar zenith angle ≥0.5 and statistically significant (details in ref. ^4^). CCI51 discrepancy: Pearson correlation between 2001–2018 FireCCILT11and FireCCI51 annual BA time series <0.5 and statistically significant (details in ref. ^5^).

While the FireCCILT11 product reduces some of the problems found in its beta-version predecessor^3^, the fixes often merely shifted the problems to different regions and spatial scales, and we reported that the FireCCILT11 product remains inconsistent in many important fire regions during much of its 37-year time series and particularly within the tropics and the United States^4^. Readers may consult our published analyses for details, but what is important here is to appreciate the spatially pervasive extent of the FireCCILT11 inconsistencies. To this end, Fig. 1 distills our recent findings^4,5^ into a global map depicting the regions where the FireCCILT11exhibits major satellite orbit-drift artifacts and/or very poor agreement with the FireCCI51 “parent” BA product that was used to train the FireCCILT11 algorithm. The FireCCI51 product^6^ was produced using high quality NASA Terra Moderate Resolution Imaging Spectroradiometer (MODIS) satellite data for 2001–2020 (during which the Terra satellite overpass did not drift).

Notably, a comparison of Fig. 1 with the relevant global maps of C2023 (cf. Figs. 2, 3, S1–S7) suggests that all six of the reported CT domains were heavily influenced by the spurious features of the FireCCILT11 time series rather than what C2023 assume, or were led to believe, was a reasonably accurate depiction of the true burned area signal. Present also in many of the C2023 correlation maps are significant spatial discontinuities, or seams, along the boundaries of several FireCCILT11 processing regions^2^. These instances are too numerous to exhaustively enumerate here but are particularly obvious in South America and Africa, where a strong temporal correlation along the FireCCILT11 processing boundaries often abruptly changes in magnitude and even sign (e.g., C2023 Fig. S1 climate teleconnections PNA, EP, ENSO, TSA; Fig. S2 SAM, AMO; Fig. S3 PNA, EA, AMO, TNA, EA; Fig. S4 PNA, EP; Fig. S5 PNA, EA; Fig. S6 AMO, EA, NAO, SAM; Fig. S7 AMO, EA, PNA, TNA; Fig. S8 EP, WP). The net result is to produce artificial seams in at least two of the C2023 CT domains (specifically, domains 3 and 4) present in South America and Africa.

We note that C2023 describe the FireCCILT11 BA product as “…the most suitable dataset…” for their study “…because the time series is long and it performs better than other global BA products in terms of small wildfire detection capacity [Shi and Touge, 2022]”. This justification is puzzling because Shi and Touge^7^ neither use nor mention the FireCCILT11 product. Moreover, at no point do they imply that FireCCILT11 might somehow perform “…better than other global BA products…” In actuality, Shi and Touge^7^ used the MODIS-based FireCCI51 burned area data set for their analysis.

In closing, the AVHRR sensors and their respective satellite platforms were not designed for fire monitoring. Ultimately, while the FireCCILT11 AVHRR based product might be suitable for some regional studies (e.g., Descals et al. ^8^), it is inappropriate to use this BA product for any large-scale or long-term study without thoroughly considering the potential impact of the product’s artifacts and inconsistencies on the analysis, and we encourage C2023 to reconsider their analysis accordingly.
